# Short‐Term Statin Therapy Induces Hepatic Insulin Resistance Through HNF4*α*/PAQR9/PPM1*α* Axis Regulated AKT Phosphorylation

**DOI:** 10.1002/advs.202403451

**Published:** 2024-07-05

**Authors:** Yijun Lin, Shuying Wang, Zixuan Li, Yuling Zhou, Ruiying Wang, Yan Wang, Yan Chen

**Affiliations:** ^1^ Xiamen Cardiovascular Hospital School of Medicine Xiamen University Xiamen 361016 China; ^2^ CAS Key Laboratory of Nutrition Metabolism and Food Safety Shanghai Institute of Nutrition and Health University of Chinese Academy of Sciences Chinese Academy of Sciences Shanghai 200031 China

**Keywords:** AKT phosphorylation, hepatic insulin resistance, progesterone and adiponectin receptor 9 (PAQR9), statins, type 2 diabetes

## Abstract

Statins, the first‐line medication for dyslipidemia, are linked to an increased risk of type 2 diabetes. But exactly how statins cause diabetes is yet unknown. In this study, a developed short‐term statin therapy on hyperlipidemia mice show that hepatic insulin resistance is a cause of statin‐induced diabetes. Statin medication raises the expression of progesterone and adiponectin receptor 9 (PAQR9) in liver, which inhibits insulin signaling through degradation of protein phosphatase, Mg^2+^/Mn^2+^ dependent 1 (PPM1*α*) to activate ERK pathway. STIP1 homology and U‐box containing protein 1 (STUB1) is found to mediate ubiquitination of PPM1*α* promoted by PAQR9. On the other hand, decreased activity of hepatocyte nuclear factor 4 alpha (HNF4*α*) seems to be the cause of PAQR9 expression under statin therapy. The interventions on PAQR9, including deletion of PAQR9, caloric restriction and HNF4*α* activation, are all effective treatments for statin‐induced diabetes, while liver specific over‐expression of PPM1*α* is another possible tactic. The results reveal the importance of HNF4*α*‐PAQR9‐STUB1‐PPM1*α* axis in controlling the statin‐induced hepatic insulin resistance, offering a fresh insight into the molecular mechanisms underlying statin therapy.

## Graphical abstract

How statins cause new‐onset diabetes is unclear. This study finds that short‐term statin therapy triggers hepatic insulin resistance. Statins inhibit HNF4*α* to activate PAQR9 expression in hepatocytes, which promote the STUB1‐mediated ubiquitination and degradation of PPM1*α* to decrease insulin response. The study offers a fresh insight into the molecular mechanisms underlying statin‐induced diabetes.

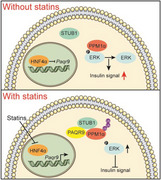

## 1 Introduction

Dyslipidemia, one of the main features of metabolic syndrome, is inextricably linked to the risk of atherosclerotic cardiovascular diseases (ASCVD).^[^ ^1^ ^]^ Triglyceride‐rich lipoproteins, particularly their cholesterol‐rich remnants, are now recognized as causal agents for ASCVD among all clinical indicators.^[^ ^2^ ^]^ Current researches support that development of atherosclerotic lesions probably requires low‐density lipoprotein (LDL),^[^ ^3^ ^]^ making it an appropriate target for drug‐based therapies to prevent coronary heart disease. According to 2019 ACC/AHA guideline on the primary prevention of cardiovascular disease, statin therapy is first‐line treatment for primary prevention of ASCVD.^[^ ^4^ ^]^ Statins are inhibitors of 3‐hydroxy‐3‐methylglutaryl‐CoA reductase (HMGCR), which directly mediates mevalonate biosynthesis and controls downstream cholesterol production. Effective statin regimens reduce LDL cholesterol by more than 50%, which lowers major cardiovascular events and coronary heart disease (CHD) death by more than 20%.^[^ ^5^ ^]^

Despite great benefits to ASCVD, numerous clinical trails reveal a variety of adverse effects of statin therapy. Statin use accompanies with increased risk of type 2 diabetes (T2D), where meta‐analysis revealed a 9% rise in the incidence of new‐onset diabetes with statin medication.^[^ ^6^ ^]^ More horrifyingly, the risk doesn't differ depending on whether a statin is rosuvastatin,^[^ ^7^ ^]^ atorvastatin^[^ ^8^ ^]^ or simvastatin.^[^ ^9^ ^]^ Some research examined the impact of statins on pancreatic cells and demonstrated that insulin secretion is attenuated by statin‐induced blockage of voltage‐gated Ca^2+^ channels in cells.^[^ ^10^ ^]^ However, recently researches indicate that taking atorvastatin causes an increase in insulin secretion,^[^ ^11^ ^]^ indicating that insulin resistance is the primary cause of statin‐induced T2D. Some researchers indicated that statins activated hepatic gluconeogenesis, which finally leads to dysglycemia.^[^ ^12^ ^]^ Blockage of the mevalonate pathway decreased prenylation might also involve inflammation or AKT phosphorylation to cause insulin resistance.^[^ ^13^, ^14^ ^]^ However, it is still unclear how statin therapy‐related increases in insulin resistance work, either the key organ of the statin‐induced insulin resistance or the detailed molecular mechanism.

PAQR9 is a progesterone and adiponectin receptor (PAQR) superfamily member that is localized to the endoplasmic reticulum (ER) and is highly expressed in the liver.^[^ ^15^, ^16^ ^]^ Despite being a member of the receptor family, PAQR9's ligand has not yet been identified, and its primary role in the ER appears to be as a scaffold protein.^[^ ^17^ ^]^ We have stated that PAQR9 functions as a liver energy sensor and controls the oxidation of fatty acids while fasting.^[^ ^16^ ^]^ As an energy sensor responsive to feeding‐fasting treatment, PAQR9 is predicted to participate in the regulation of glucose homeostasis. Here, we report that besides fatty acid metabolism, hepatic PAQR9 is a key regulator of hepatic insulin sensitivity in response to statin therapy. We show that short‐term statin treatment activates the hepatic expression of PAQR9 to mediate decreased AKT phosphorylation through the regulation of PPM1*α* stability, and inhibition of PAQR9 or overexpression of PPM1*α* could promote hepatic insulin signaling under statins.

## 2 Results

### 2.1 Short‐Term Statin Treatment Leads to Dysglycemia and Hepatic Insulin Resistance

**Figure 1:**
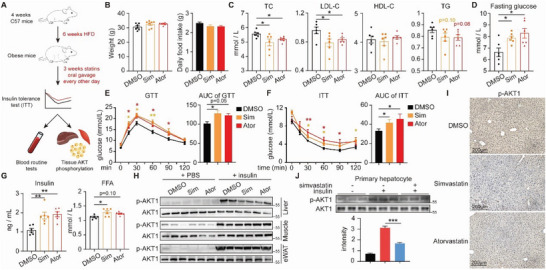
Short‐term statin treatment leads to dysglycemia and hepatic insulin resistance. A) A diagram for the short‐term statin treatment mouse model. B) Body weight (left) and average food intake (right) of the mice treated with DMSO, simvastatin or atorvastatin (n = 7 for each group. Sim, simvastatin; Ator, atorvastatin.). C,D) The levels of TC, LDL‐C, HDL‐C, TG (C) and fasting glucose (D) in the serum of the mice. n = 6 for each group. E,F) GTT (E) and ITT (E) of the mice. n = 7 for each group. G) The levels of insulin (left) and FFA (right) in the serum of the mice. n = 6 for each group. H) Western blotting to detect AKT1 S473 phosphorylation (p‐AKT1) in the liver, skeletal muscle, and eWAT of the mice at the end of the experiment. I) Representative images of p‐AKT1 staining of liver sections of the DMSO, simvastatin and atorvastatin‐treated mice. J) Western blotting to detect AKT1 phosphorylation in the DMSO or simvastatin‐treated mouse primary hepatocytes. Quantification of p‐AKT1 relative to AKT1 is shown in the below panel. All the quantitative data were analyzed with one‐way ANOVA and are shown as mean ±SEM. ^*^ *p* < 0.05; ^**^ *p* < 0.01; ^***^ *p* < 0.001.

We first designed a short‐term statin‐treated model with a 3‐week statins oral gavage regimen followed by 6–8 weeks of HFD to assess the lipid‐lowering effect (**Figure** **1**A). Both 10 mg kg^−1^ simvastatin and 20 mg kg^−1^ atorvastatin had no effect on food consumption or body weight (Figure 1B), but both treatments reduced total cholesterol (TC), triglycerides (TG), and low‐density lipoprotein cholesterol (LDL‐C) while having no significant effects on high‐density lipoprotein cholesterol (HDL‐C) (Figure 1C). However, we noticed that statin‐treated mice had significantly higher fasting glucose levels than untreated mice (Figure 1D), which was usually reported in long‐term model. Both glucose tolerance and insulin tolerance was aggravated by both simvastatin and atorvastatin (Figure 1E,F). The fact that statins generate diabetes was further corroborated by higher levels of blood insulin and free fatty acids (FFA), two hyperinsulinemia indices linked to insulin resistance (Figure 1G), indicating that short‐term statin therapy could lead to pre‐diabetes.

In insulin‐sensitive tissues, insulin resistance can emerge on its own and be the result of many causes.^[^ ^18^ ^]^ We detected AKT phosphorylation in liver, muscle and epididymal white adipose tissue (eWAT), three prominent insulin‐responding tissues. However, only the liver showed considerable insulin resistance (Figure 1H), also verified by IHC staining (Figure 1I). The phenotype is also observed in isolated primary hepatocytes and HepG2 cells in vitro (Figure 1J; Figure S1B‐D, Supporting Information), demonstrating that hepatic insulin resistance is directly induced by statins.

### 2.2 PAQR9 is Increased by Statins and it Affects Hepatic Insulin Resistance Under Statin Treatment In Vitro

**Figure 2:**
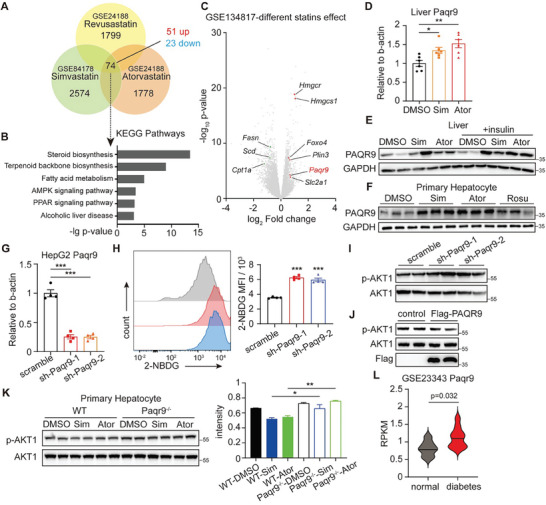
PAQR9 is increased by statins and it aggravates hepatic insulin resistance under statin treatment. A) Venn diagram showing changes of transcripts with a rigorous criteria of absolute log_2_FC≥2 and p‐value<0.05. The data were from GEO datasets GSE24188 and GSE84178. B) KEGG analysis of the 74 synchronously altered genes in (A). C) Volcano plot of GSE134817 to show gene expression changes after different statins treatment. D) Relative mRNA levels of *Paqr9* in the liver of the mice as in Figure 1 (n = 6 for each group). E) Western blotting of PAQR9 in the liver of the mice as in Figure 1. F) Western blotting of PAQR9 in mouse primary hepatocytes under the treatment of DMSO, simvastatin (Sim), atorvastatin (Ator) and rosuvastatin (Rosu). G) Relative mRNA levels of *Paqr9* in HepG2 to detect the efficiency of *Paqr9* knockdown with sh‐RNA. H) Flow cytometry analysis of 2‐NBDG uptake of the HepG2 cells as in (G) under simvastatin treatment. Mean fluorescence intensity was shown in the right panel (n = 4 for each group). I,J) Western blotting to detect p‐AKT1 in the HepG2 cells knockdown (I) or overexpressed (J) PAQR9 with simvastatin treatment and insulin stimulation. K) Western blotting to detect p‐AKT1 in mouse primary hepatocytes from WT or *Paqr9* ^−^ *^/−^* mouse, under simvastatin or atorvastatin treatment and insulin stimulation. Quantification of p‐AKT1 relative to AKT1 is shown in the right panel. L) The expression of *Paqr9* in the liver of normal and diabetic patients. The data were from GEO datasets GSE23343. All the quantitative data except (L) were analyzed with one‐way ANOVA and were shown as mean ±SEM, data of (L) was analyzed with student t‐test. ^*^ *p* < 0.05; ^**^ *p* < 0.01; ^***^ *p* < 0.001.

To identify the major regulator of statin‐induced hepatic insulin resistance, we examined some public sequencing datasets of statin‐treated hepatocytes (GSE24188 and GSE84178). Only 74 synchronously altered genes were chosen under various statin treatment conditions (**Figure** **2**A). KEGG enrichment analysis of these 74 genes showed 6 significant pathways involved in statin‐induced phenotypes (Figure 2B), where AMPK and PPAR signaling were shown substantial associations. To further verify our findings, another dataset GSE134817, which referred to general statins effect on hepatocytes, was cited (Figure 2C). A series of genes belonging to AMPK or PPAR pathways were significantly changed under statin treatment, indicating the powerful effect on these signals.

A highly expressed protein in the liver known as PAQR9 has been linked to these two pathways,^[^ ^16^ ^]^ which is also shown in the volcano plot (Figure 2C). In mouse livers treated with simvastatin or atorvastatin, we discovered elevated levels of *Paqr9* mRNA and protein (Figure 2D,E). The up‐regulation was also seen in isolated hepatocytes (Figure 2F), demonstrating that statin therapy can directly promote hepatic PAQR9.

The PAQR9‐knockdown HepG2 cells were then generated (Figure 2G). Under statin therapy, the PAQR9‐reduced cells displayed increased glucose uptake and insulin sensitivity than control cells (Figure 2H,I; Figure S2C, Supporting Information), similar to the results from the *Paqr9* ^−/−^ hepatocytes (Figure 2K). Whereas, overexpression of PAQR9 weakened AKT phosphorylation (Figure 2J). Additionally, through the public RNA‐seq dataset of liver, hepatic *Paqr9* expression is higher in diabetic people (Figure 2L), showing that PAQR9 is an insulin sensitivity reducer in hepatocytes.

### 2.3 PAQR9 Ablation Ameliorates Statin‐Induced Diabetes

**Figure 3:**
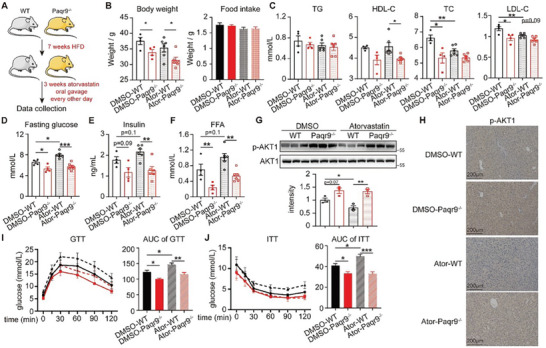
Deletion of PAQR9 ameliorates statin‐induced insulin resistance. A) A diagram for the atorvastatin‐treatment WT or *Paqr9* ^−^ *^/−^* mouse model. B) Body weight (left) and average food intake (right) of the mice in (A). C) TG, TC, HDL‐C and LDL‐C in the serum of the mice as in (A). D–F) The levels of fasting glucose (D), insulin (E) and fatty acids (F) in the serum of the mice as in (A). For (B–F), n = 4 for DMSO‐WT and DMSO‐*Paqr9* ^−^ *^/^* ^−^ mice; n = 6 for Ator‐WT and Ator‐*Paqr9* ^−^ *^/^* ^−^ mice. G) Western blotting to detect hepatic AKT1 phosphorylation in WT and *Paqr9* ^−^ *^/^* ^−^ mice as in (A). Quantification of p‐AKT1 relative to AKT1 is shown in the below panel. H) Representative images of p‐AKT1 staining of liver sections of the mice as in (A). I,J) GTT (I) and ITT (J) of the mice as in (A). n = 4 for DMSO‐WT and DMSO‐*Paqr9* ^−^ *^/^* ^−^ mice; n = 6 for Ator‐WT and Ator‐*Paqr9* ^−^ *^/^* ^−^ mice. All the data were analyzed with one‐way ANOVA and were shown as mean ±SEM. ^*^ *p* < 0.05; ^**^ *p* < 0.01; ^***^ *p* < 0.001.

With the *Paqr9* ^−/−^ mice have been reported,^[^ ^16^ ^]^ we explored whether deletion of PAQR9 protected mice from atorvastatin‐induced hepatic insulin resistance (**Figure** **3**A). *Paqr9* ^−/−^ mice were thinner than wild type mice (Figure 3B) and showed better cholesterol metabolism (Figure 3C). Importantly, atorvastatin‐raised levels of fasting glucose, insulin and free fatty acids were seemed much less in *Paqr9* ^−/−^ mice (Figure 3D‐F), which showed attenuated diabetes in knockout mice. Similar effects were also found in GTT and ITT (Figure 3I,J). Hepatic AKT phosphorylation in *Paqr9* ^−^ *^/^* ^−^ animals was generally increased (Figure 3G,H), demonstrating improvement in hepatic insulin resistance.

We have reported that *Paqr9* is an energy sensitive gene in the liver, which sharply decreased during fasting.^[^ ^16^ ^]^ Furthermore, according to our prior RNA‐sequencing results, either caloric restriction (CR) or a fasting‐mimicking diet (FMD) lowers the expression of hepatic *Paqr9* (unpublished observations). Therefore, we designed an atorvastatin combined CR or intermittent fasting (IF) model to detect whether these diet programs could alleviate statin‐induced diabetes (Figure S3A, Supporting Information). Both CR and IF reduced the amounts of hepatic PAQR9 protein (Figure S3H, Supporting Information), also, drastically reduced fasting glucose levels together with insulin and FFA in serum (Figure S3E,F, Supporting Information). Moreover, insulin tolerance was also ameliorated consistently by dietary intervention, especially the hepatic insulin signaling (Figure S3G‐I, Supporting Information), indicating a practicable intervention on the statin side effect. Overall, PAQR9 deficiency reverses statin‐induced hepatic insulin resistance and diabetes.

### 2.4 PAQR9 Regulates Insulin Sensitivity Through PPM1*α*

**Figure 4:**
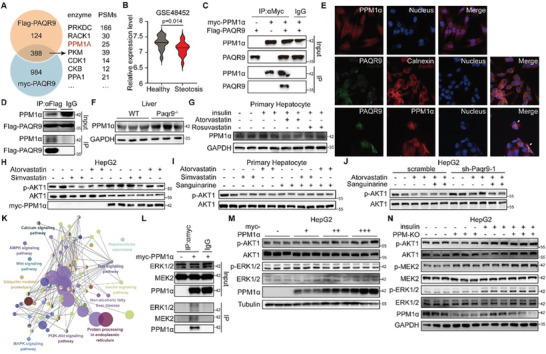
PAQR9 attenuates hepatic insulin sensitivity through inhibition of PPM1*α*‐ERK1/2 pathway. A) Venn diagram showing the proteins interacted with Flag‐ and Myc‐tagged PAQR9 by AP‐MS in HepG2 cells. The top kinases or phosphatases were shown in the right. B) The expression of *Ppm1a* in the liver of normal and hepatic steotosis patients. The data were from GEO datasets GSE48452 and analyzed with Student t test. C,D) Co‐IP assays to analyze the interaction between PPM1*α* and PAQR9. For (C), HEK293 cells were transfected with Myc‐tagged PPM1*α* and Flag‐tagged PAQR9, followed by IP with anti‐Myc. For (D), HepG2 cells were transfected with Flag‐tagged PAQR9 followed by IP with anti‐Flag. E) Representative images of immunofluorescent (IF) staining to detect the localization of GFP‐PAQR9 and Myc‐PPM1*α* in HepG2 cells. The white arrows pointed overlapping parts. F) Western blotting to detect PPM1*α* in the liver of WT or *Paqr9* ^−^ *^/^* ^−^ mouse. G) Western blotting to detect PPM1*α* in mouse primary hepatocytes under atorvastatin or rosuvastatin treatment with 30 min insulin stimulation. H) Western blotting to detect AKT1 phosphorylation in PPM1*α*‐overexpressed HepG2 cells under atorvastatin or simvastatin treatment with 30 min insulin stimulation. I) Western blotting to detect AKT1 phosphorylation in mouse primary hepatocytes under atorvastatin or simvastatin and sanguinarine treatment with 30 min insulin stimulation. J) Western blotting to detect AKT1 phosphorylation in *Paqr9* knockdown HepG2 cells under atorvastatin and sanguinarine treatment with 30 min insulin stimulation. K) Network diagram showing KEGG analysis of the proteins interacted with Myc‐PPM1*α*. The proteins were detected by AP‐MS in HepG2 cells. L) Co‐IP assay to analyze interaction of PPM1*α* with endogenous ERK1/2 and MEK2 in HEK293T cells. M) Western blotting to detect AKT1 and ERK1/2 phosphorylation in HepG2 cells overexpressed with different amount of Myc‐PPM1*α*. Cells were stimulated with insulin for 30 min. N) Western blotting to detect AKT1, MEK2 and ERK1/2 phosphorylation in control (Ctrl) and PPM1*α*‐deleted (PPM‐KO) HepG2 cells with or without 30 min insulin stimulation.

PAQR9 is an ER‐localized scaffold protein in our earlier investigation.^[^ ^17^ ^]^ We used affinity capture mass spectrometry (AP‐MS) to find proteins interacted with Flag‐ and Myc‐tagged PAQR9 to better understand how PAQR9 functions in the insulin signaling pathway (**Figure** **4**A). 388 proteins were pulled down by both tagged PAQR9 in HepG2 cells, and 21 of those proteins had phosphatase or kinase annotations. We focused that PPM1*α*, known as protein phosphatase 2C alpha (PP2C*α*), was one of the highest‐ranking phosphatases (Figure 4A). It's interesting to note that hepatic PPM1*α* appears to be linked to diabetes and liver steotosis (Figure 4B; Figure S4A, Supporting Information).

We employed co‐immunoprecipitation (co‐IP) to validate the interaction of PAQR9 and PPM1*α* and found Flag‐PAQR9 interacted with exogenous and endogenous PPM1*α* in the assay (Figure 4C,D; Figure S4B, Supporting Information). PAQR9 and PPM1*α* were partially co‐localized in ER in HepG2 cells (Figure 4E), further indicating the interaction of these two proteins. Notably, *Paqr9* ^−/−^ liver and fasting mouse liver showed enhanced PPM1*α* expression (Figure 4F; Figure S3H, Supporting Information), and PPM1*α* levels dropped when treated with statins (Figure 4G), suggesting a negative correlation with PAQR9. We measured the insulin sensitivity under statin treatment to determine whether PPM1*α* is involved in the control of insulin sensitivity. As shown in Figure 4H,I, AKT phosphorylation was reversed by PPM1*α* overexpression but further decreased by PPM1*α* inhibitor sanguinarine upon statin treatment. PAQR9‐effects on insulin sensitivity could be rescued by PPM1*α* (Figure 4J; Figure S4C, Supporting Information), revealing that PPM1*α* was downstream of PAQR9 in the insulin signaling pathway.

### 2.5 PPM1*α* Regulates Insulin Sensitivity Through ERK1/2 Phosphorylation

Although it has been shown that PPM1*α* regulates the insulin signaling pathway, the consequences and mechanisms differ greatly depending on the organ.^[^ ^19^, ^20^, ^21^, ^22^ ^]^ We employed AP‐MS with Myc‐PPM1*α*, and 1088 interacted proteins were used for KEGG enrichment analysis. The pathways FDR < 0.001 were shown with a network diagram (Figure 4K), where the core pathways were PI3K‐AKT signal and AMPK signal. ERK1, ERK2, and MEK2 were observed in MS, and PPM1*α* could bring all of them down in a Co‐IP assay (Figure 4L), consistent with previous reports.^[^ ^23^ ^]^ As another pathway responding to insulin, MAPK signal was long‐termly indicated to negatively regulate insulin sensitivity by balancing the PI3K/AKT pathway.^[^ ^24^, ^25^ ^]^ To investigate the relationship among ERK, AKT, and PPM1*α*, we detected phosphorylated AKT and ERK1/2 in PPM1*α*‐overexpressed HepG2 cells. Therefore, overexpression of PPM1*α* reduced ERK1/2 phosphorylation but increased AKT1 phosphorylation in a dose‐dependent manner (Figure 4M), whereas PPM1*α* deletion increased ERK1/2 phosphorylation under insulin stimulation (Figure 4N). As a result, these findings suggested that PPM1*α* enhanced insulin signaling by balancing ERK and AKT pathways in hepatocytes.

### 2.6 PAQR9 Mediates PPM1*α* Ubiquitination and Degradation by STUB1

**Figure 5:**
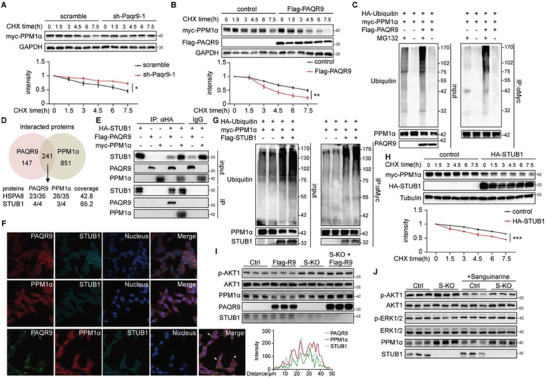
PAQR9 mediates poly‐ubiquitination and degradation of PPM1*α* by interaction with STUB1. A,B) Western blotting to detect protein stability of PPM1*α* with *Papr9* knockdown (A) and overexpression (B). HepG2 cells with *Paqr9* knockdown or overexpression were transiently transfected with Myc‐PPM1*α* and treated with CHX for various times, followed by immunoblotting with the antibodies as indicated. Quantitation of the results from three independent experiments is shown in the below panel. C) Poly‐ubiquitination of PPM1*α* in HepG2 cells with PAQR9 overexpression. HepG2 cells were transiently transfected HA‐Ubiquitin, Myc‐PPM1*α* and Flag‐PAQR9, and then treated with MG132 for 6 h, followed by IP and immunoblotting. D) Venn diagram showing the proteins interacted with PAQR9 and Myc‐PPM1*α* by AP‐MS in HepG2 cells. E) Co‐IP assay to analyze interaction of PPM1*α*, PAQR9 and STUB1. HepG2 cells were transfected with Myc‐tagged PPM1*α*, Flag‐tagged PAQR9 and HA‐tagged STUB1, followed by IP with anti‐HA. F) Representative images of IF staining to detect the localization of Flag‐ or GFP‐PAQR9, Myc‐PPM1*α* and HA‐STUB1 in HepG2 cells. The white arrows pointed overlapping parts. The right panel shows plots of signal intensity (y axis) against distance in mm (x axis) to indicate occurrence of overlaps of the three proteins. G) Poly‐ubiquitination of PPM1*α* in HepG2 cells with STUB1 overexpression. H) Western blotting to detect protein stability of PPM1*α* with STUB1 overexpression. Quantitation of the results from three independent experiments is shown in the below panel. I) Western blotting to detect AKT1 phosphorylation and PPM1*α* in HepG2 cells with Flag‐PAQR9 overexpressed (Flag‐R9) and STUB1 knockout (S‐KO). Cells were stimulated with insulin for 30 min. J) Western blotting to detect AKT1 and ERK1/2 phosphorylation and PPM1*α* in control (Ctrl) or STUB1 knockout (S‐KO) HepG2 cells with or without sanguinarine treatment. Cells were stimulated with insulin for 30 min. All the curves were analyzed with two‐way ANOVA and the points were shown as mean ±SEM. ^*^ *p* < 0.05; ^**^ *p* < 0.01; ^***^ *p* < 0.001.

We then concentrated on PAQR9's control over PPM1*α*‐mediated phosphorylation of AKT. Figure 4F illustrates that deletion of PAQR9 resulted in a rise in PPM1*α* protein levels, which was also observed in CR or IF mice (Figure S3H, Supporting Information). We observed the stability of PPM1*α* in PAQR9‐deleted or overexpressed HepG2 cells to determine whether PAQR9 influenced the degradation of PPM1*α*. PAQR9‐deletion prolonged PPM1*α*’s half‐life in HepG2 cells (**Figure** **5**A), but overexpression sped up the rate of PPM1*α* degradation (Figure 5B). PAQR9 was reported to regulate protein stability through the ubiquitin‐proteasome pathway,^[^ ^16^, ^17^ ^]^ and we discovered that PAQR9 overexpression promoted poly‐ubiquitination of PPM1*α* (Figure 5C), indicating PAQR9 promotes the degradation of PPM1*α* protein via an ubiquitin‐proteasome dependent pathway.

We discovered 241 proteins interacting with both PPM1*α* and PAQR9 based on the results of AP‐MS, 11 of which are E3 ligases (Figure 5D). One of the co‐interacted ligases is STUB1, also known as CHIP, and its chaperone HSPA8 was also identified. Co‐IP assays confirmed the interaction between STUB1 and the other two proteins (Figure 5E; Figure S5A,B, Supporting Information). The co‐localization of the three proteins in HepG2 cells was identified by an immunofluorescence experiment (Figure 5F). Importantly, overexpression of STUB1 enhanced PPM1*α* ubiquitination and accelerated its degradation (Figure 5G,H), demonstrating that STUB1 is an E3 ligase for PPM1*α*.

To further explore whether PAQR9 mediates the degradation of PPM1*α* by STUB1, we detected PPM1*α* and insulin signal in STUB1‐deleted HepG2 cells. As shown in Figure 5I, STUB1 deletion raised PPM1*α* protein levels and enhanced AKT phosphorylation in HepG2. PPM1*α* was markedly reduced by PAQR9 overexpression, which disappeared in STUB1 mutant cells (Figure 5I), indicating that STUB1 is required for PAQR9‐mediated PPM1*α* degradation. Additionally, sanguinarine was able to attenuate STUB1 deletion‐increased insulin sensitivity (Figure 5J), showing PPM1*α* was a downstream target of STUB1. Furthermore, STUB1 expression was not significantly influenced by statin therapy in vivo and in vitro (Figure S5E,F, Supporting Information), indicating that statin‐accelerated PPM1*α* degradation was mediated by PAQR9 but not directly by STUB1. Overall, these findings showed that STUB1‐mediated PPM1*α* degradation is the mechanism through which PAQR9 suppresses the PPM1*α*‐ERK1/2‐AKT pathway.

### 2.7 HNF4*α* is a Negative Transcript Factor for PAQR9 in Response to Statins

**Figure 6:**
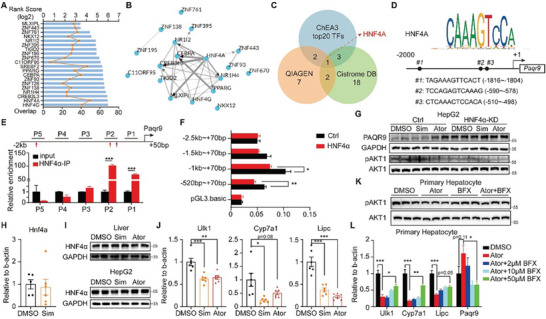
HNF4*α* is a negative transcript factor for PAQR9 in response to statins. A) The top 20 predicted transcription factors of differentially expressed genes under atorvastatin treatment. The gene expression data were from GEO datasets GSE24188 and the TF prediction was by ChEA3. The bar showed rank score and the line showed the number of overlapped genes. B) Network diagram showing the relationship between the 20 TFs as in (A). C) Venn diagram showing the TFs of *Paqr9* predicted by QIAGEN, Cistrome DB and the analysis in (A). D) Human HNF4*α* motif analysis and potential binding sites at the *Paqr9* promoter. E) ChIP‐qPCR with different regions of *Paqr9* promoter. P1 to P5 were five different segments covering the region of −2,000 bp to the transcription start site (TSS) region. The red arrows pointed the predicted binding sites of HNF4*α*. F) Dual‐luciferase reporter assay. Different lengths of putative Paqr9 promoter were cloned into luciferase‐containing plasmid and transiently expressed in the HepG2 cells along with HNF4*α*‐expressing plasmid. G) Western blotting to detect AKT1 phosphorylation and PAQR9 in control (Ctrl) or HNF4*α* knockdown (HNF4*α*‐KD) HepG2 cells with DMSO, simvastatin (Sim) or atorvastatin (Ator) treatment. Cells were stimulated with insulin for 30 min. H) Relative mRNA levels of *Hnf4a* in the liver of the mice as in Figure 1. n = 6 for each group. I) Western blotting to detect HNF4*α* in the liver of the mice as in Figure 1 (up) and in HepG2 cells treated with simvastatin or atorvastatin (below). J) Relative mRNA levels of *Ulk1, Cyp7a1* and *Lipc* in the liver of the mice as in Figure 1. n = 6 for each group. K) Western blotting to detect AKT1 phosphorylation and PAQR9 in mouse primary hepatocytes with atorvastatin (Ator) and benfluorex (BFX). L) Relative mRNA levels of *Paqr9, Ulk1, Cyp7a1* and *Lipc* in mouse primary hepatocytes with atorvastatin (Ator) and different concentration of benfluorex (BFX). The results of ChIP‐qPCR and luciferase assay were analyzed with Student t test and the mRNA level of (J and L) were analyzed with one‐way ANOVA. All data were shown as mean ±SEM. ^*^ *p* < 0.05; ^**^ *p* < 0.01; ^***^ *p* < 0.001.

We have discussed the PAQR9‐STUB1‐PPM1*α* axis in the control of insulin signaling, however, how statin treatment induces PAQR9 expression is still unclear. We used ChEA3 (https://maayanlab.cloud/chea3/) to evaluate the transcription factors of differentially expressed genes in order to investigate the primary transcriptional alterations caused by statin treatment. In the top 20 ranking TFs, we found certain key factors including HNF4*α*, NR1H4, MLXIPL, and NR1I2 (**Figure** **6**A,B). Interestingly, HNF4*α* is also one of the predicted PAQR9 TFs from both QIAGEN and Cistrome DB (Figure 6C). Three putative HNF4*α* binding sites in the human *Paqr9* promoter region were examined by JASPAR (Figure 6D). Then, using ChIP‐qPCR, we verified that HNF4*α* could bind to the human *Paqr9* promoter directly at predicted sites #2 and #3 (Figure 6E).

We examined the promoter activity of the *Paqr9* gene using a dual‐luciferase reporter experiment in HepG2 cells to confirm if HNF4*α* was involved in the control of *Paqr9* gene expression. The luciferase activity was alleviated by HNF4*α* overexpression (Figure 6F), indicating a negative regulation of HNF4*α* on *Paqr9* expression, especially on the −1k/+70 bp segment. Consistently, overexpression of HNF4*α* decreased the expression of *Paqr9* in HepG2 cells (Figure S6B, Supporting Information), while knockdown of HNF4*α* elevated PAQR9 protein level with or without statin treatment (Figure 6G). We also detected the regulating condition in mice. Even though the homology of *Paqr9* upstream sequences seemed not very high between human and mouse (Figure S9A, Supporting Information), mouse HNF4*α* was predicted to bind to three sites similar to which was shown in HepG2 cells (Figure S9B, Supporting Information). ChIP‐qPCR further proved that mHNF4*α* could also directly bind to the antisense strand of the Paqr9 promoter (Figure S9C, Supporting Information), just like the results in human cells.

We found *Paqr9* gene expression was increased under statin treatment (Figure 2). However, neither the mRNA nor the protein of HNF4*α* was altered by statins either in HepG2 cells or mouse liver (Figure 6H,I). We next analyzed the downstream targets of HNF4*α*. Various targets, including *Ulk1*, *Cyp7a1*, and *Lipc*, were all downregulated under statin treatment in vivo (Figure 6J), indicating the function inhibition of HNF4*α* by statins, consistent with the increase in expression of *Paqr9*. By molecular docking simulation, we found that both simvastatin and atorvastatin could directly bind with HNF4*α* (Figure S10, Supporting Information), which might be the cause of the inhibition.

Mutations in the HNF4*α* gene have been linked to the risk of T2D and maturity‐onset diabetes of the young (MODY).^[^ ^26^ ^]^ In addition, in HFD mice, ablation of hepatic HNF4*α* results in insulin resistance.^[^ ^27^ ^]^ In our experiments, we found HNF4*α* knockdown impaired AKT phosphorylation regardless of statin treatment (Figure 6G). Overexpression of HNF4*α* or its activator, benfluorex, were shown to partially rescue statin effects in either HepG2 or mouse primary hepatocytes (Figure 6K,L; Figure S6B,C, Supporting Information). In light of the aforementioned research, statins may cause hepatic insulin resistance by inhibiting HNF4*α*.

### 2.8 Benfluorex‐Combined Treatment or Liver‐Specific Overexpression of PPM1*α* are Potential Therapies for Short‐Term Statin‐Induced Diabetes

**Figure 7:**
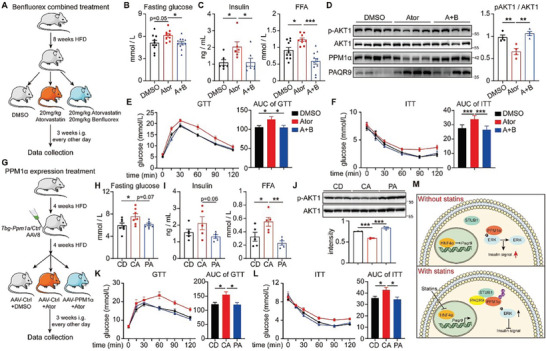
Benflurex‐combined treatment or liver‐specific overexpression of PPM1*α* are potential therapies for statin‐induced hepatic insulin resistance. A) A diagram for the benfluorex combined atorvastatin‐treatment mouse model. B,C) The levels of fasting glucose (B), insulin and FFA (C) in the serum of the mice as in (A). D) Western blotting to detect AKT1 phosphorylation, PPM1*α* and PAQR9 in the liver of the mice as in (A). Quantification of p‐AKT1 relative to AKT1 is shown in the right panel. E,F) GTT (E) and ITT (F) of the mice as in (A). For (B–F) n = 10 for group DMSO; n = 9 for group Ator; n = 11 for group A+B. G) A diagram for the liver specific PPM1*α* overexpression combined atorvastatin‐treatment mouse model. H,I) The levels of fasting glucose (H), insulin and FFA (I) in the serum of the mice as in (G). J) Western blotting to detect AKT1 phosphorylation in the liver of the mice as in (G). Quantification of p‐AKT1 relative to AKT1 is shown in the below panel. K,L) GTT (K) and ITT (L) of the mice as in (G). For (H–L), n = 7 for each group. M) A proposed model depicts PAQR9 regulation on statin‐induced insulin resistance through the STUB1/ PPM1*α*/ERK axis. All the quantitative data were analyzed with one‐way ANOVA and were shown as mean ±SEM. ^*^ *p* < 0.05; ^**^ *p* < 0.01; ^***^ *p* < 0.001.

As we highlighted in Figure 6, HNF4*α* is a potential target for insulin resistance brought on by statins, so we tried to combine benfluorex and atorvastatin for treatment (**Figure** **7**A). Benfluorex used to be a clinical diabetes medicine by activating HNF4*α* (Figure S7A, Supporting Information), which also improved levels of insulin and FFA affected by atorvastatin treatment, as well as fasting glucose (Figure 7B,C). Both glucose tolerance and insulin tolerance were alleviated by the drug combination (Figure 7E,F), and hepatic AKT phosphorylation was increased by benfluorex (Figure 7D; Figure S7H, Supporting Information). Notably, benfluorex might likewise lower the PAQR9 levels raised by atorvastatin (Figure 7D), corroborating the in vitro pathway we looked at (Figure 6).

Another target might be used for statin‐induced diabetes intervention is PPM1*α*, as discussed in Figure 4. With the AAV‐8 mediated liver‐specific PPM1*α* overexpression mice (Figure 7G; Figure S8A, Supporting Information), we found that fasting glucose was reduced compared with the control mice (Figure 7H), as well as insulin and FFA (Figure 7I). Hepatic AKT phosphorylation was strongly upregulated by PPM1*α* overexpression whereas p‐ERK1/2 was attenuated (Figure 7J; Figure S8D,F, Supporting Information), corresponding with the signaling pathways found in vitro (Figure 4). Hepatic PPM1*α* overexpression also significantly improved both GTT and ITT in the mice (Figure 7K,L), consistent with the above index. What is more interesting is that PPM1*α* overexpression could further improve the lowering cholesterol effect of atorvastatin (Figure S8E, Supporting Information), suggesting a great potency for clinical transformation. Above all, we demonstrated that different interventions on HNF4*α*‐PAQR9‐PPM1*α* pathways could be effective therapies for statin‐induced diabetes.

## 3 Discussion

While statins are broadly and long‐termly used in the clinical treatment of dyslipidemia, few studies have focused on their potential for causing new‐onset diabetes. In this article, we present evidence that PAQR9 responds to statins in the liver to control hepatic insulin resistance. Short‐term statin therapy may considerably increase hepatic insulin resistance, as observed in cultured hepatocytes. As an energy sensor and lipid‐oxidative regulator in hepatocytes, PAQR9 is found to be upregulated by statin treatment, while *Paqr9* ^−^ *^/^* ^−^ mice exhibit ameliorated insulin resistance under statins or HFD. Mechanistically, PAQR9 functions as a scaffold to engage with STUB1 and PPM1*α*, leading to the proteasome‐dependent degradation of PPM1*α*. We demonstrate that PPM1*α* is a positive regulator of insulin‐AKT signaling in hepatocytes because it dephosphorylates ERK1/2, which explains how PAQR9 affects hepatic insulin signaling. Additionally, we identified PAQR9's upstream regulator and discovered that HNF4*α* is a negative regulator of PAQR9, whose activity is suppressed when statins are used (Figure 7M). Some approaches, including inhibition of PAQR9 and activation of PPM1*α* or HNF4*α*, could improve hepatic insulin signaling under statin treatment. Consequently, HNF4*α*‐PAQR9‐PPM1*α* pathway is crucial for the regulation of statin‐induced hepatic insulin resistance.

As the most recommended drugs for ASCVD, statins have a remarkable impact on lowering LDL‐C supported by strong evidence.^[^ ^6^ ^]^ The debate over statin side‐effects, however, has never been resolved. Statin‐associated muscular symptom (SAMS) is a common reason for quitting statin therapy because they occur in 10–29% of people receiving the medication.^[^ ^6^, ^28^ ^]^ But several clinical studies viewed that long‐term statin therapy caused few symptomatic adverse events, which did not appear to fundamentally change how statins were used.^[^ ^5^, ^29^ ^]^ How to balance the benefits and harms of statin therapy is an important proposition for all cardiovascular doctors. The theory of statin‐induced new‐onset diabetes may interfere with peripheral insulin signaling and pancreatic *β*‐cell function, while the insulin resistance might be the core factor. However, neither the affected organs nor the insulin resistance signaling pathway have been precisely identified. Adipose tissue inflammation,^[^ ^13^ ^]^ hepatic autophagy^[^ ^12^ ^]^ and myocyte glucose absorption^[^ ^10^ ^]^ were all believed to play a role in the process. Moreover, most studies focus on long‐term statin therapy, but whether short‐term treatment mediates metabolic disorder is little understood. As we highlight in this study, a 3‐week model could develop hepatic insulin resistance, emphasizing the cautious attitude toward statin use.

Interestingly, a recent study indicates a substantial link between statin use and liver disease protection.^[^ ^30^ ^]^ Statin use reduced the development of liver diseases by 15%, especially liver cell carcinoma, by over 40%. Numerous studies have demonstrated the therapeutic efficacy of statins in HCC outside of clinical meta‐analysis.^[^ ^31^, ^32^ ^]^ Inflammation, angiogenesis or MYC signal might be the targets of statins for cancer therapy. It's worth noting that the hyper‐activating PI3K/AKT pathway is associated with aggressive hepatocellular carcinoma,^[^ ^33^ ^]^ and atorvastatin has been reported to decrease proliferation and invasiveness of hepatocytes by AKT signal.^[^ ^34^ ^]^ In our investigation, we found statins downregulated hepatic insulin signaling, further indicating that statins potential to prevent cancer may involve inhibiting AKT phosphorylation. By the way, according to the pathway, PAQR9 might play an anti‐cancer factor in the liver, which has been verified in our lab (unpublished observation).

As previously reported, PAQR9 is a highly expressed ER scaffold protein in the liver.^[^ ^16^ ^]^ The regulation of fatty acid oxidation in liver by PAQR9 was discovered, and in this article we demonstrated that PAQR9 modified the hepatic insulin signal. These studies suggest that PAQR9 is an important metabolic regulator in liver. *Paqr9* is primarily expressed in hepatocytes (Figure S2, Supporting Information); however, we have recently revealed that this gene also functions in the pancreas to control glucose homeostasis.^[^ ^35^ ^]^ More interestingly, PAQR9 altered dyslipidemia in addition to insulin sensitivity without statin treatment (Figure 3), indicating that PAQR9 has complicated metabolic activity. We have also noticed the function of PAQR9 in regulating HFD‐induced diabetes and NAFLD, which might relate to its effect on lipid metabolism (unpublished observation). Further research is being done on the tissue‐specific intervention on PAQR9 and the other regulatory mechanisms.

HNF4*α*, a well‐known transcription factor for liver development and function, regulates the expression of several genes involved in lipid, glucose, and drug metabolism. We discovered that HNF4*α* was a negative regulator of Paqr9, which is the first time that HNF4*α*’s direct transcription‐inhibitory role has been reported. Importantly, HNF4*α* is one of the most critical hepatic TFs in response to statins (Figure 6A,B), and statins reduced HNF4*α* activity but not expression by directly bind to the protein (Figure S10, Supporting Information). This is the first report that HNF4*α* is a statin target, and the active group of statins, 3,5 ‐dihydroxycarboxylic acid, could bind to the regulatory domains of both human and mouse HNF4*α* with high affinity (Figure S10, Supporting Information). These results suggest that this transcription factor can be broadly targeted by different statins. However, further experimental evidence is needed to confirm the conclusions in the future.

HNF4*α* plays a key role in maturity‐onset diabetes of the young (MODY1).^[^ ^36^ ^]^ A recent study showed liver‐specific knockdown of HNF4*α* significantly damaged glucose homeostasis,^[^ ^27^ ^]^ which highlighted the glucose metabolic function of hepatic HNF4*α*, consistent with our research. On the other hand, even though benfluorex showed an improving effect on insulin signaling in our detection, the reducing‐lipid impact of atorvastatin appeared to be lessened when benfluorex was added to the treatment regimen (Figure S7B‐D, Supporting Information). Considering that HNF4*α* controls hepatic and plasma lipid metabolism,^[^ ^37^ ^]^ inhibition of HNF4*α* might also be a mechanism of statin‐induced hypolipidemia. Another question is that benfluorex has been reported to cause heart valve hyperplasia and pulmonary hypertension, which leads to the prohibition of its clinical use. Luckily, those side effects were not observed after a short‐term treatment (Figure S7E,F, Supporting Information). However, the practical application should wait until a securer agonist is found.

The other two proteins in the signaling system, PPM1*α* and STUB1, have been reported to mediate glucose homeostasis. PPM1*α* seems to perform different functions in different cells, which is a positive regulator of insulin sensitivity in adipocytes^[^ ^21^ ^]^ but is also reported as an AMPK inhibitor in other cells.^[^ ^20^ ^]^ Notably, PPM1*α*‐mediated dephosphorylation of SGK2 has been reported to involve in simvastatin‐induced gluconeogenesis,^[^ ^38^ ^]^ suggesting an adjuvant but indirect inhibition of glucose homeostasis. Our research supported PPM1*α*’s beneficial role in the liver by showing how it regulates hepatic insulin signaling. PPM1*α* overexpression in the liver showed great improvement in insulin sensitivity under statin treatment and further lipid metabolism (Figure 7), suggesting that liver‐specific PPM1*α* overexpression could be used as a treatment for insulin resistance or even other metabolic syndromes. STUB1 has been shown to regulate diabetes through the degradation of INSR,^[^ ^39^ ^]^ and we demonstrated another mechanism to control insulin signaling. It appears that STUB1 modulates insulin sensitivity partly through the PPM1*α*‐ERK‐AKT pathway because STUB1 deletion caused an increase in PPM1*α* (Figure 5J), which could be reduced by sanguinarine. This is the first report of STUB1 mediating proteasome‐dependent degradation of PPM1*α*, revealing a novel E3 ligase for PPM1*α*. On the other hand, statins were reported to develop the ubiquitin‐proteasome system in muscle, which is now thought to be a possible mechanism of statin‐associated myopathies.^[^ ^40^ ^]^ Our results supported that PAQR9 associated proteasome system was activated in the liver, however, no significant changes in STUB1 were observed, revealing a different regulatory pattern of statins in liver.

In summary, the results of our study indicate that PAQR9 functions as a key mediator of hepatic insulin signaling under statin use. Therefore, interventions on HNF4*α*‐PAQR9‐STUB1‐PPM1*α* pathway can be a promising strategy to break the link between statins and type 2 diabetes. Activation of HNF4*α* by benfluorex and overexpression of PPM1*α* might be potential methods for statin side‐effects, while dietary interventions can provide greater benefits. Our results, we contend, point to a deeper comprehension of the role of hepatic metabolism in the management of diabetes.

## 4 Experimental Section

### Reagents and Plasmids

The following primary antibodies were used: anti‐Myc (1:2000 for immunoblotting and 1:200 for Co‐IP, Abclonal, AE009), anti‐Flag (1:4000 for immunoblotting and 1:200 for Co‐IP, Abclonal, AE005), anti‐HA (1:2000 for immunoblotting and 1:200 for Co‐IP, Abclonal, AE008), anti‐PAQR9 (1:1000 for immunoblotting and 1:200 for IHC, sigma, HPA052798), anti‐PPM1*α* (1:1000, Abclonal, A6699), anti‐STUB1 (1:1000, Abclonal, A11715), anti‐HNF4*α* (1:1000 for immunoblotting and 1:100 for ChIP, Abclonal, A20865), anti‐GAPDH(1:20000, Abclonal, AC033), anti‐*β*‐Tubulin (1:3000, ZSGB‐Bio, TA‐10), anti‐AKT1 (1:1000, Abclonal, A17909), phospho‐S473‐AKT1 (1:500 for immunoblotting and 1:200 for IHC, Abclonal, AP0637), anti‐ERK1/2 (1:1000, Abclonal, A16686), anti‐phospho‐ERK1/2 (1:1000, Abclonal, AP0974), anti‐MEK2 (1:1000, Abclonal, A0253), anti‐phospho‐MEK2‐T394 (1:1000, Abclonal, AP0121). The HRP‐conjugated secondary antibodies were purchased from Abclonal (AS014 and AS003, 1:3000). Alexa Fluor 488/ 546/ 647 conjugated secondary antibodies were purchased from Invitrogen (A11001, A11030 and A31571, 1:100).

The following drugs used were purchased from MCE: Atorvastatin (HY‐17379), simvastatin (HY‐17502), rosuvastatin (HY‐17504), sanguinarine (HY‐N0052), benfluorex (HY‐B1058). 2‐NBDG was purchased from APExBio (B6035) and insulin was from Procell Life Science&Technology (PB180432).

Vectors including pLVX‐Myc/HA/Flag, HA‐tagged ubiquitin, different tagged‐PAQR9, Lenti‐CRISPRV2, pGL.3‐basic with various lengths of *Paqr9* promoter, pRL‐TK and *Paqr9* shRNA constructs were reported previously.^[^ ^16^ ^]^ PPM1*α* and STUB1 were cloned from the cDNA of HepG2 cells by PCR and respectively inserted into vector pLVX‐Myc and pLVX‐HA. PPM1*α*, STUB1 and HNF4*α* knockout plasmid was generated by CRISPR‐Cas9 system with Lenti‐CRISPRV2 containing sequences of sgRNA: PPM1*α*: 5′‐GCAACCCATTACCCTGCCCC‐3′; STUB1: 5′‐GTTGGGGATGAGCTGTTCCT‐3′; HNF4*α*: 5′‐GGCACCGTAGTGTTTGCCCG‐3′.

### Cell Culture, Transfection and Cell Line Construction

HEK293T cells, HepG2 cells and mouse primary hepatocytes were cultured in high‐glucose DMEM (4.5 g L^−1^) with 100 units mL^−1^ penicillin/streptomycin and 10% FBS at 37 °C with 5% CO2. Mouse primary hepatocytes were isolated from male mice using collagenase perfusion, seeded and cultured on collagen‐coated plates in the medium. HEK293T cells were transfected with PEI, and HepG2 cells were transfected with Lipo3000. *Paqr9*‐knockdown, PPM1*α*‐deleted, STUB1‐deleted and HNF4*α*‐knockdown HepG2 cells were generated by lentivirus transfection and screened with puromycin. Lentivirus was packaged with the psPAX2‐pMD2.G system. In brief, each 10‐mm dish of HEK293T cells was transfected with 7.5 mg of psPAX2, 3 mg of pMD2.G, and 10 mg of shRNA/ sgRNA‐containing plasmids. The cell culture medium was collected 48 and 72 h later and directly used to infect HepG2 cells for 48 h with 4 mg mL^−1^ polybrene, then treated with 1.5 mg mL^−1^ puromycin for 24 h.

For statins treatment, HepG2 cells were seeded in polylysine‐coated plates overnight. Then, 5 mmol L^−1^ simvastatin or 10 mmol L^−1^ atorvastatin/ rosuvastatin were added for 24–48 hours. For insulin treatment, cells were washed twice with PBS and treated with 100 ng mL^−1^ insulin for 30 min.

### Protein Extraction, Co‐IP and Immunoblotting

Cultured cells were lysed in RIPA buffer with complete protease inhibitors and phosphatase inhibitors (Roche), and the supernatant was collected after centrifugation at 4 °C for 10 min at 12 000 rpm. For tissue extract, the mouse tissues were cut and grinded and lysed in the RIPA buffer, and then the tissue suspension was centrifuged at 12 000 rpm min^−1^ for 15 min to collect the supernatant for immunoblotting. For ubiquitination assay, the cells were treated with or without 10 mmol L^−1^ MG132 for 6 h to block proteasomal degradation of proteins before lysis. To analyze protein degradation rate, the cells were treated with cycloheximide (CHX) for various times.

Co‐IP was performed 36 h after transfection. Cells were washed three times with PBS and then lysed with RIPA as above for 15 min at 4 °C, then the homogenates were centrifuged for 10 min at 12 000 rpm at 4 °C. Then, 10% of the supernatant was harvested for immunoblotting as inputs, while the remaining cell lysate was incubated with the indicated antibodies overnight at 4 °C. Protein A/G plus agarose beads (Beyotime Biotechnology, Shanghai, China, P2055) were added to the supernatants for another 3 h at 4 °C. After extensive washing with PBS, the beads were spun down and resuspended, followed by immunoblotting.

The proteins were eluted with SDS sample buffer (LABLEAD, Beijing, China, G2527) and assayed by western blotting using 10–12% SDS‐PAGE gels and PVDF membranes.

### Animal Studies

The *Paqr9* ^−^ *^/^* ^−^ mice were reported previously.^[^ ^16^ ^]^ All animals were maintained and used in accordance with the guidelines of the Institutional Animal Care and Use Committee of the Xiamen University (approval no. XMULAC20230163). Mice were maintained on a 12 h light/dark cycle at 25 °C. Only male mice were used in the study, and all of the mice were on C57/BL6J background. All data from animal experiments were filtered by average±2S.D.

For HFD‐induced obesity, mice were fed an HFD (60% of kilocalories from fat, cat. no. D12492; Ready Dietech) starting from 5 weeks of age. Weights of food and mice were observed every week, and statin treatment started when average mice weight over 30 g. Mice were randomly assigned to each group. Glucose tolerance test (GTT) (with 1 g kg^−1^ glucose i.p.) and insulin tolerance test (ITT) (with 1 unit kg^−1^ insulin i.p.) were performed at 4th week after statins treatment. The blood glucose level was determined with blood glucose test strips (Bayer) at 0, 15, 30, 60, 90, and 120 min after injection for both GTT and ITT.

The dietary intervention started together with atorvastatin treatment. The mice of group DH (DMSO with HFD) and AH (atorvastatin with HFD) were fed with HFD ad libitum. For group AC (atorvastatin with caloric restriction), the mice were fed with HFD at 70% of the group AH. For group AF (atorvastatin with intermittent fasting), the mice were fed for 3 days with HFD at 35% of the group AH, followed by HFD ad libitum for 4 days.

Mice were administered with adeno‐associated virus (AAV) vectors via tail vein injection for overexpressing PPM1*α* in the livers when the mice have been fed with HFD for 4 weeks. The AAV used was packaged and purified by HanBio, Shanghai. These AAVs harbor the thyroxine binding globulin (TBG) promoter that can perform the liver‐specific overexpression of PPM1*α* in mice.

For detection of fasting glucose and insulin, mice were fasted for 16 h and the blood was taken from the tip of the tails. As for detection of insulin‐stimulated AKT phosphorylation in tissues, mice fasted for 6 h were injected with a dose of insulin (4 units kg^−1^) and the tissues were collected 8 min later. Basal level of AKT phosphorylation was detected with tissues from mice injected with saline. The levels of TG, TC, HDL‐C, LDL‐C and free fatty acids (FFA) were detected with serum from mice fasted for 6 h. The FFA and insulin were detected by ELISA. The kit for FFA was purchased from Nanjing Jiancheng Bioengineering Institute (A042‐1‐1) and the kit for insulin detection was purchased from Crystal Chem (90080). Other indexes were detected by biochemistry analyzer.

### Mass Spectrometry

Affinity purified proteins were collected by Co‐IP as above, then proteins were reduced, alkylated, and loaded onto an SDS‐PAGE gel to remove any detergents and LCMS incompatible reagents. The gel plugs were then excised, digestion and analyzed with LCMS by Shanghai Applied Protein Techenology Co., Ltd.

### Immunohistochemistry and Immunofluorescent Staining

Mouse livers were cut and fixed with 4% PFA for 24 h for paraffin‐embedding. Hematoxylin‐ eosin staining and IHC for PAQR9 and p‐AKT1 were performed by Servicebio (Wuhan, China). The mounted slides were imaged with an EVOS FL AUTO2 microscope. For immunofluorescent staining, HepG2 cells were cultured on glass coverslips and transfected with different plasmids. At 24 h after transfection, the cells were fixed with paraformaldehyde for 10 min, permeabilized with 0.1% Triton X100 in PBS for 1 min. The cells were blocked in 3% bovine serum albumin in PBS overnight at 4°C, then were incubated with primary antibodies and secondary antibodies for 2 h respectively. The nuclei were stained with Hoechst 33342. Fluorescence images were acquired with Nikon AX with NSPARC.

### RNA Isolation and RT‐qPCR

Total RNA of cells was lysed by TRIzol reagent (Invitrogen, Carlsbad, CA, U.S.A.) and purified according to the manufacturer's instructions. RNA was reverse‐transcribed with Hifair AdvanceFast 1st Strand cDNA Synthesis Kit (Yeasen, Shanghai, China) to obtain cDNA. Real‐time PCR was performed with Genious SYBR Green Fast qPCR Mix (Abclonal, Shanghai, China).

### Dual‐luciferase Reporter Assay

HepG2 cells were co‐transfected with a Flag‐tagged HNF4*α*, pRL‐TK expressing renilla‐luciferase, and pGL.3‐basic under the control of different lengths of the putative promoter region of the Paqr9 gene, with a ratio of 1:0.05:1. 24 h after the transfection, the cells were lysed and then used to analyze luciferase activity using a kit purchased from Yeasen (Shanghai, China,11402ES60).

### ChIP‐qPCR

HepG2 cells or mouse primary hepatocytes were digested and fixed with 1% (wt/vol) formaldehyde for 10 min, subsequently following by quenching with glycine. After centrifugation at 1700×g for 5 min, the cells were resuspended with lysis buffer (50 mmol L^−1^ HEPES, 140 mmol L^−1^ NaCl, 1 mmol l^−1^ EDTA, 20% glycerol, 0.5% NP40 and 0.25% Triton X‐100) for 10 min, and then washed twice respectively with washing buffer (10 mmol L^−1^ HEPES, 200 mmol L^−1^ NaCl, 1 mmol L^−1^ EDTA and 0.5 mmol L^−1^ EGTA) and shearing buffer (10 mmol L^−1^ Tris‐HCl, 1 mmol L^−1^ EDTA and 0.1% SDS) to collect nucleus. The chromatin of nuclear pellets was sonicated to generate 200‐ to 1000‐bp fragments with Bioruptor (Diagenode minichiller 300). After sonication, the supernatant was collected and washed with conversion buffer (10 mmol L^−1^ Tris‐HCl, 280 mmol L^−1^ NaCl, 1 mmol L^−1^ EDTA, 1 mmol L^−1^ EGTA, 0.25% Triton X‐100, 0.2% sodium deoxycholate and 0.1% SDS). Supernatant was incubated with dynabeads for one hour for pre‐clearance, with 5% as input and other 95% incubating with HNF4*α*‐coated beads overnight. The beads were washed with RIPA buffer, high salt buffer and TE, followed by elution of DNA by fresh elution buffer containing RNase. Eluted DNA was incubated in 65° for 6 h for de‐crosslinking. DNA was purified with DNA concentration kit (Tiangen, Beijing, China). In the ChIP‐qPCR analyses, the values from the immunoprecipitated samples were normalized to those from the input DNA.

### Molecular Docking

The crystal structures of the human and mouse HNF4*α* were downloaded from RCSB PDB Database (https://www.pdbus.org), and the structures of simvastatin and atorvastatin were downloaded from PubChem (https://pubchem.ncbi.nlm.nih). Ligands and water removal of proteins were handled by PyMOL. OutDock Tools 1.5.6 was used for hydrogen addition, amino acid optimization and active pocket mocking. Outdock Vina 1.1.2 was used for docking, while PyMOL was used to visualize the docking results.

### Statistics

All data were shown as mean ± SEM. Statistical analyses were performed using Graph Prism 9.5 software. Quantitation results were analyzed with Student t test, one‐way ANOVA or two‐way ANOVA as figure legend. Values of *p* < 0.05 were considered statistically significant.

## Conflict of Interest

The authors declare no conflict of interest.

## Author Contributions

Y.L. and Y.C. designed the experiments. Y.L. performed the experiments and data analysis. Y.L. wrote the manuscript and prepared the figures. Z.L., S.W., Y.Z., and R.W. provided assistance for animal experiments. S.W., Y.W., and Y.C. provided comments. Y.C. supervised the study and provided writing revision. All authors read and approved the manuscript. Y.L. is the guarantor of this work and takes responsibility for the integrity of the data and the accuracy of the data analysis.

## Supporting information

Supporting Information

## Data Availability

The data that support the findings of this study are available from the corresponding author upon reasonable request.
