# Supplementary material for: Short‐Term Statin Therapy Induces Hepatic Insulin Resistance Through HNF4α/PAQR9/PPM1α Axis Regulated AKT Phosphorylation
Source: Adv Sci (Weinh). 2024 Jul 5;11(34):2403451. doi: 10.1002/advs.202403451 (PMC11425881; doi:10.1002/advs.202403451)
Supplement: Supplementary file 1 — Supporting Information [file ADVS-11-2403451-s001.pdf]

ADVANCED SCIENCE
Open Access

# Supporting Information

for *Adv. Sci.*, DOI 10.1002/advs.202403451

Short-Term Statin Therapy Induces Hepatic Insulin Resistance Through HNF4α/PAQR9/PPM1α Axis Regulated AKT Phosphorylation

*Yijun Lin*, Shuying Wang, Zixuan Li, Yuling Zhou, Ruiying Wang, Yan Wang and Yan Chen**

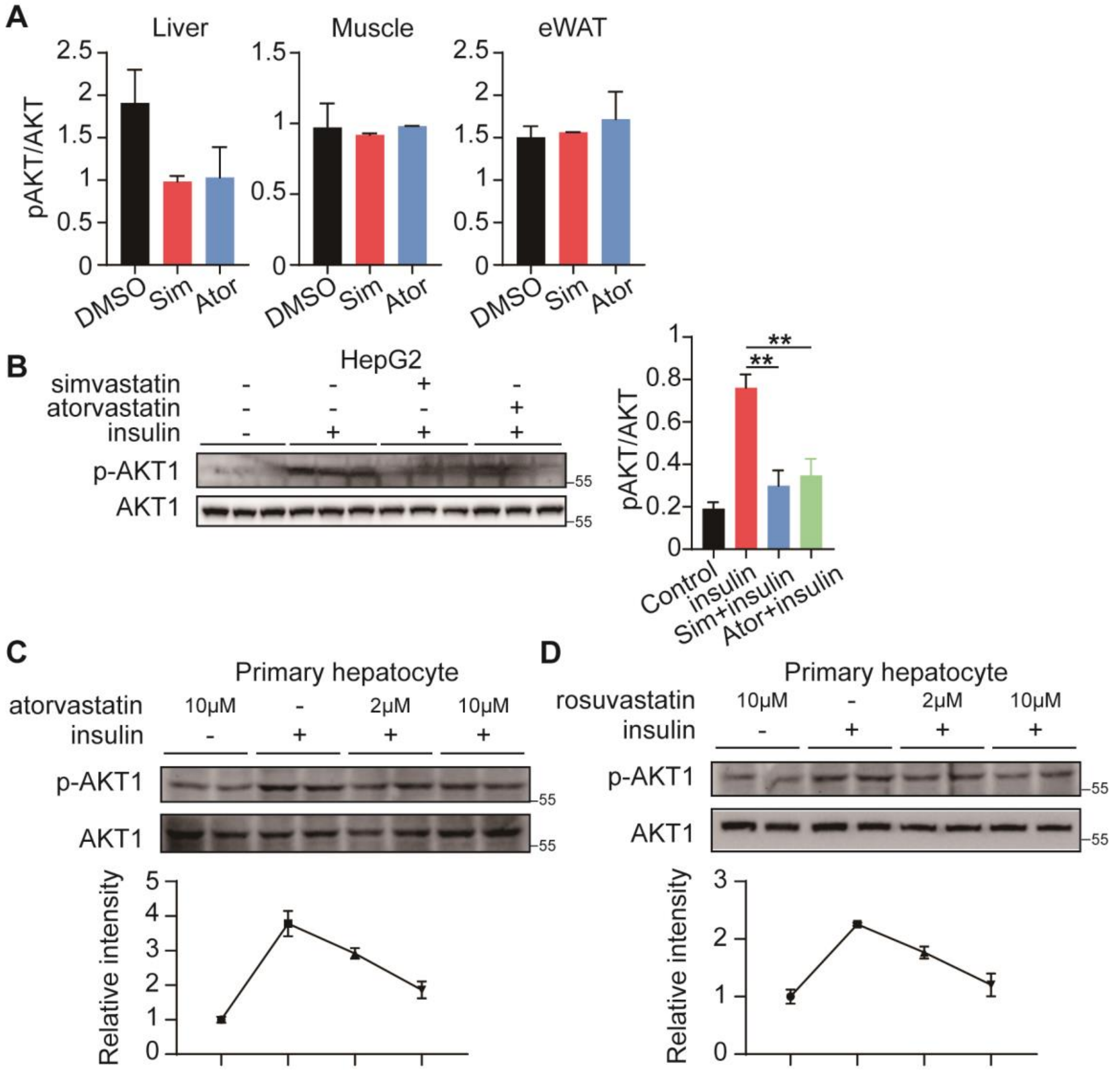


**Figure S1. Short-term statins therapy induced hepatic insulin resistance.**

**(A)** Quantification of phosphorylated AKT1 relative to total AKT1 of **Figure 1H**.

**(B)** Western blotting to detect AKT1 phosphorylation in control, simvastatin or atorvastatin treated HepG2 cells. Quantification of p-AKT1 relative to AKT1 is shown in the right panel.

**(C-D)** Western blotting to detect AKT1 phosphorylation in control, atorvastatin (**C**) or rosuvastatin (**D**) treated primary hepatocytes. Quantification of p-AKT1 relative to AKT1 is shown in the below panel.

All the quantitative data were analyzed with one-way ANOVA and are shown as mean ±SEM. ***P < 0.001.

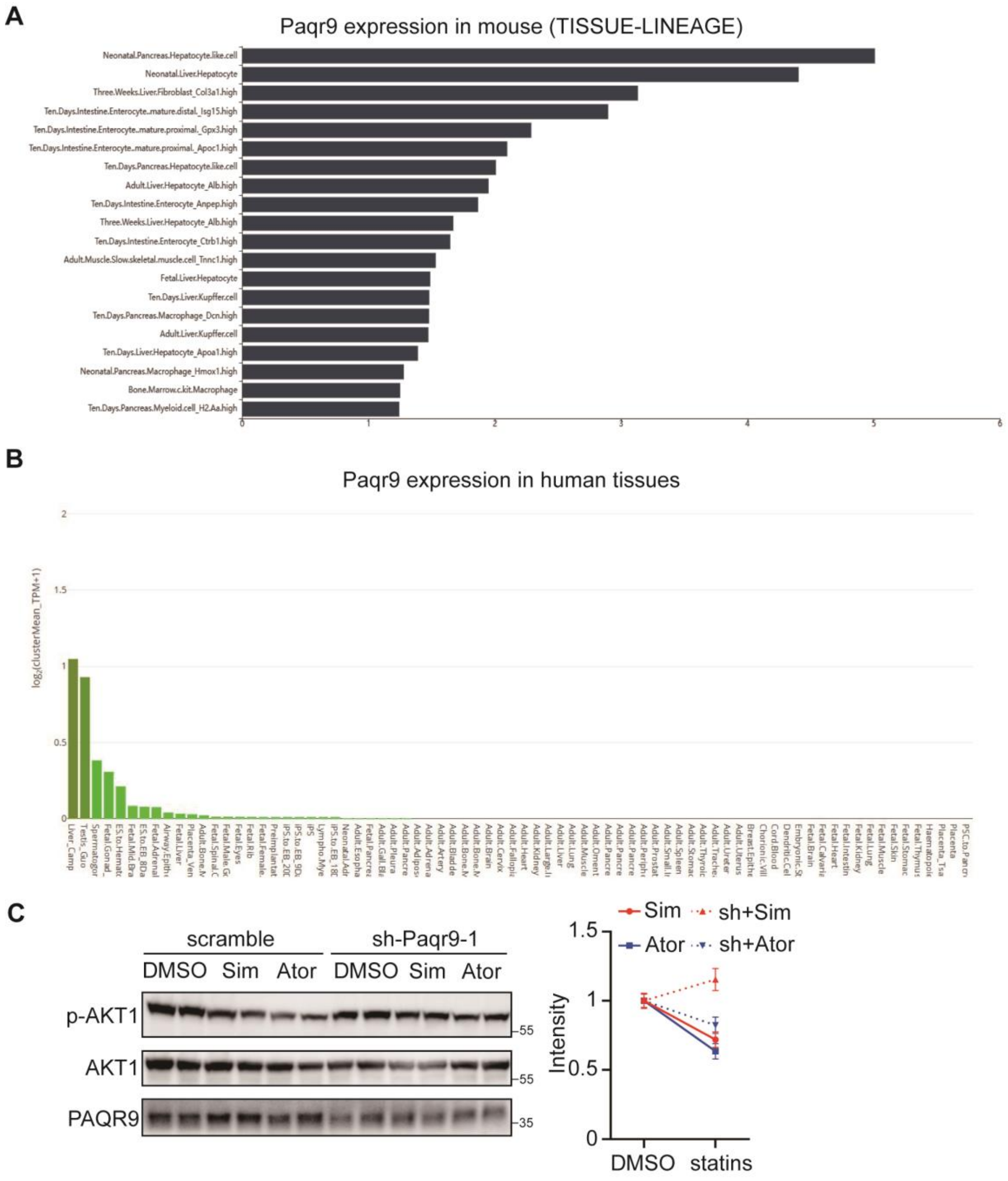


**Figure S2.** ***Paqr9*** **expression in different tissues and was increased by statins.**

**(A-B)** *Paqr9* expression in mouse tissues **(A)** and human tissues **(B)** by single cell RNA-sequence. The data respectively come from Mouse Cell Atlas (https://bis.zju.edu.cn/MCA/index.html) and Human Cell Landscape (https://bis.zju.edu.cn/HCL/).

**(C)** Western blotting to detect p-AKT1 in the control and *Paqr9*-knockdown HepG2 cells under simvastatin or atorvastatin treatment with 30min insulin stimulation. Quantification of p-AKT1 relative to AKT1 is shown in the right panel.

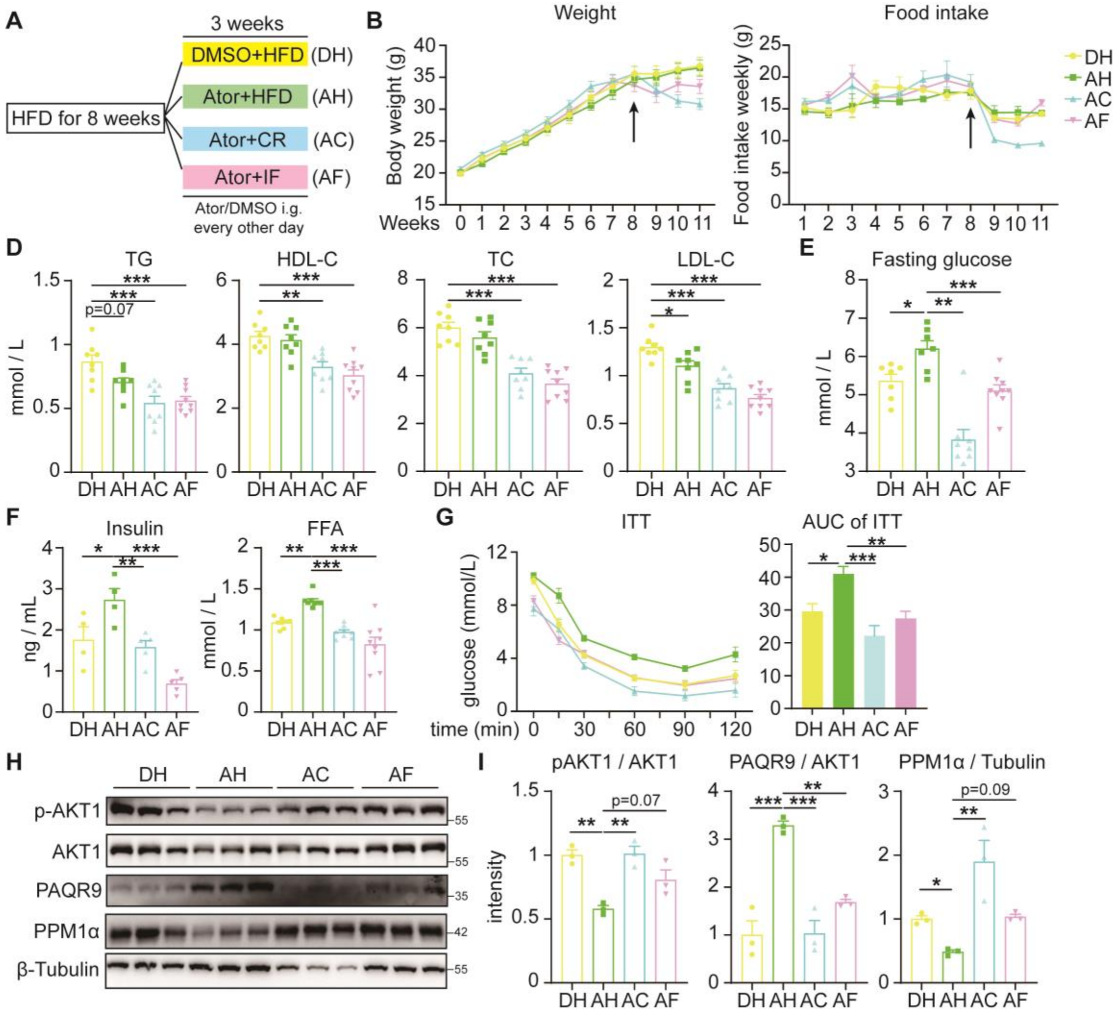


**Figure S3. Dietary intervention ameliorate statin-induced insulin resistance.**

**(A)** A diagram for the atorvastatin-treatment combined dietary intervention mouse model.

**(B-C)** Body weight **(B)** and average food intake **(C)** of the mice in **A**.

**(D)** TG, TC, HDL-C and LDL-C in the serum of the mice as in **A**.

**(E-F)** The levels of fasting glucose **(E)**, insulin and fatty acids **(F)** in the serum of the mice as in **A**.

**(G)** GTT of the mice as in **A**.

For **B-G**, n=8 for group DMSO-HFD (DH), group Atorvastatin-HFD (AH) and group Atorvastatin-caloric restriction (AC), n=9 for Atorvastatin-intermittent fasting (AF).

**(H)** Western blotting to detect hepatic AKT1 phosphorylation, PAQR9 and PPM1α in the liver of the mice.

**(I)** Quantification of the western blotting as in **H**.

All the data were analyzed with one-way ANOVA and were shown as mean ±SEM. *P < 0.05; **P < 0.01; ***P < 0.001.

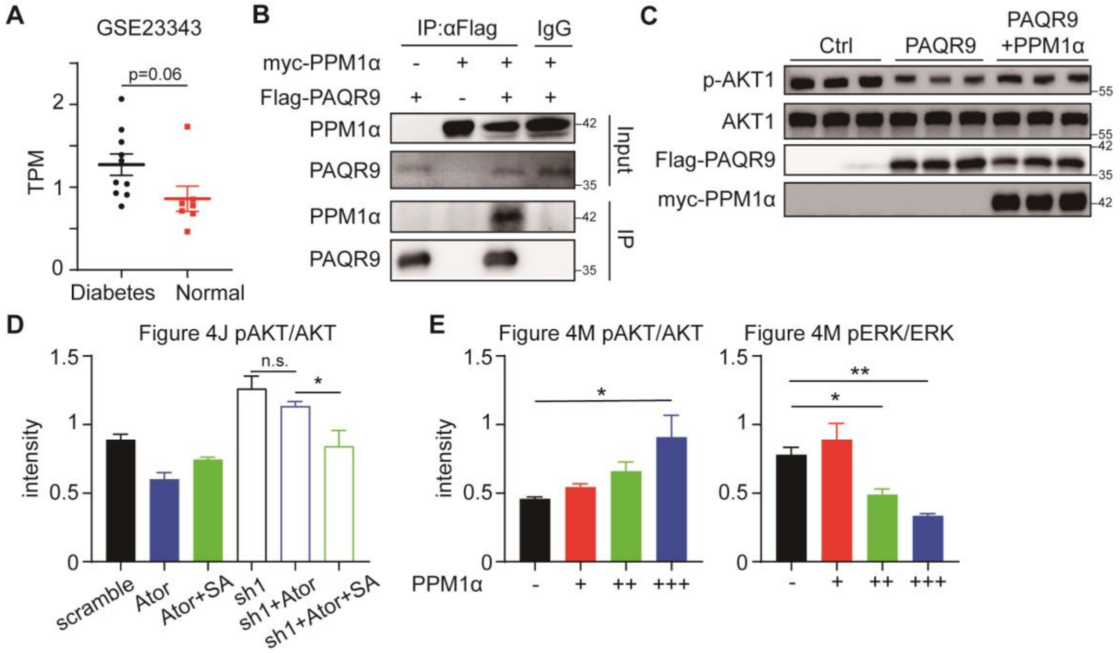


**Figure S4. PPM1α involved in PAQR9 regulated hepatic insulin sensitivity.**

**(A)** The expression of *Ppm1a* in the liver of normal and diabetic patients. The data were from GEO datasets GSE23343 and data was analyzed with Student t test.

**(B)** Co-IP assay to analyze interaction of PPM1α with PAQR9. HEK293 cells were transfected with Myc-tagged PPM1α and Flag-tagged PAQR9, followed by IP with anti-Flag.

**(C)** Western blotting to detect AKT1 phosphorylation in HepG2 cells transfected with Flag-PAQR9 and Myc-PPM1α, following by 30min insulin stimulation.

**(D-E)** Quantification of the western blotting as in **Figure 4J** and **4M**. Data were analyzed with one-way ANOVA and were shown as mean ±SEM. *P < 0.05; **P < 0.01.

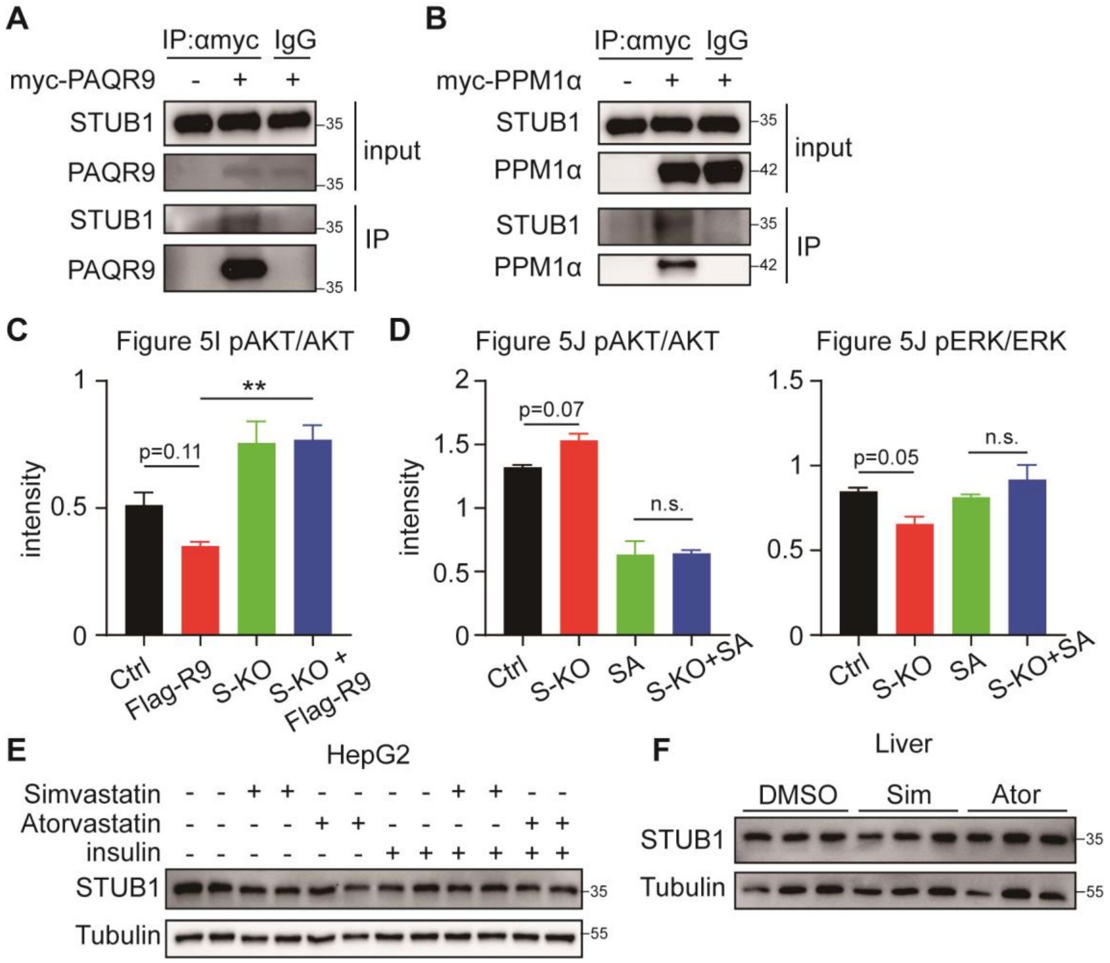


**Figure S5. STUB1 interacted with PAQR9 and PPM1α to regulate hepatic insulin sensitivity.**

**(A-B)** Co-IP assay to analyze interaction of endogenous STUB1 with Myc-PAQR9 (**A**) and Myc-PPM1α (**B**). HEK293 cells were transfected with Myc-tagged PPM1α or PAQR9, followed by IP with anti-Myc.

**(C-D)** Quantification of the western blotting as in **Figure 5I** and **5J**. Data were analyzed with one-way ANOVA and were shown as mean ±SEM. *P < 0.05; **P < 0.01.

**(E-F)** Western blotting to detect STUB1 expression in HepG2 cells (**E**) and mouse liver as in **Figure 1** (**F**).

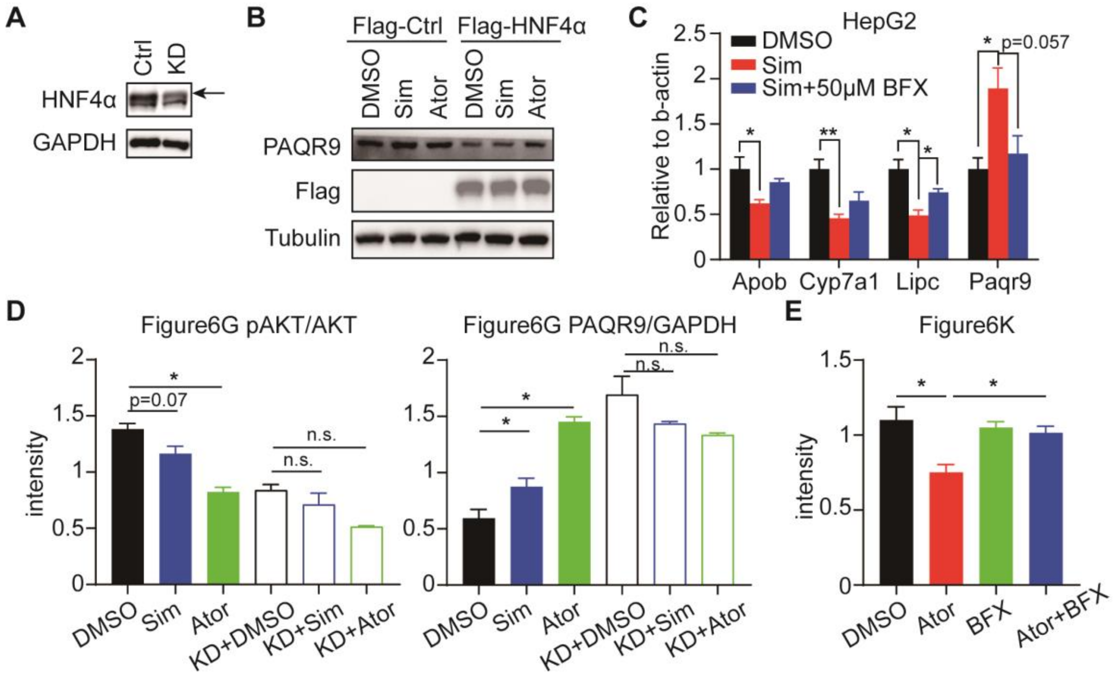


**Figure S6. HNF4α activation partially reversed statin effects.**

**(A)** Western blotting to detect the efficacy of HNF4α knockdown.

**(B)** Western blotting to detect PAQR9 expression in HNF4α overexpressed HepG2 cells with or without statins.

**(C)** Relative mRNA levels of *Paqr9, Apob, Cyp7a1* and *Lipc* in HepG2 cells with Simvastatin (Sim) and benfluorex (BFX).

**(D-E)** Quantification of the western blotting as in **Figure 6G** and **6K**. Data were analyzed with one-way ANOVA and were shown as mean ±SEM. $*P < 0.05$.

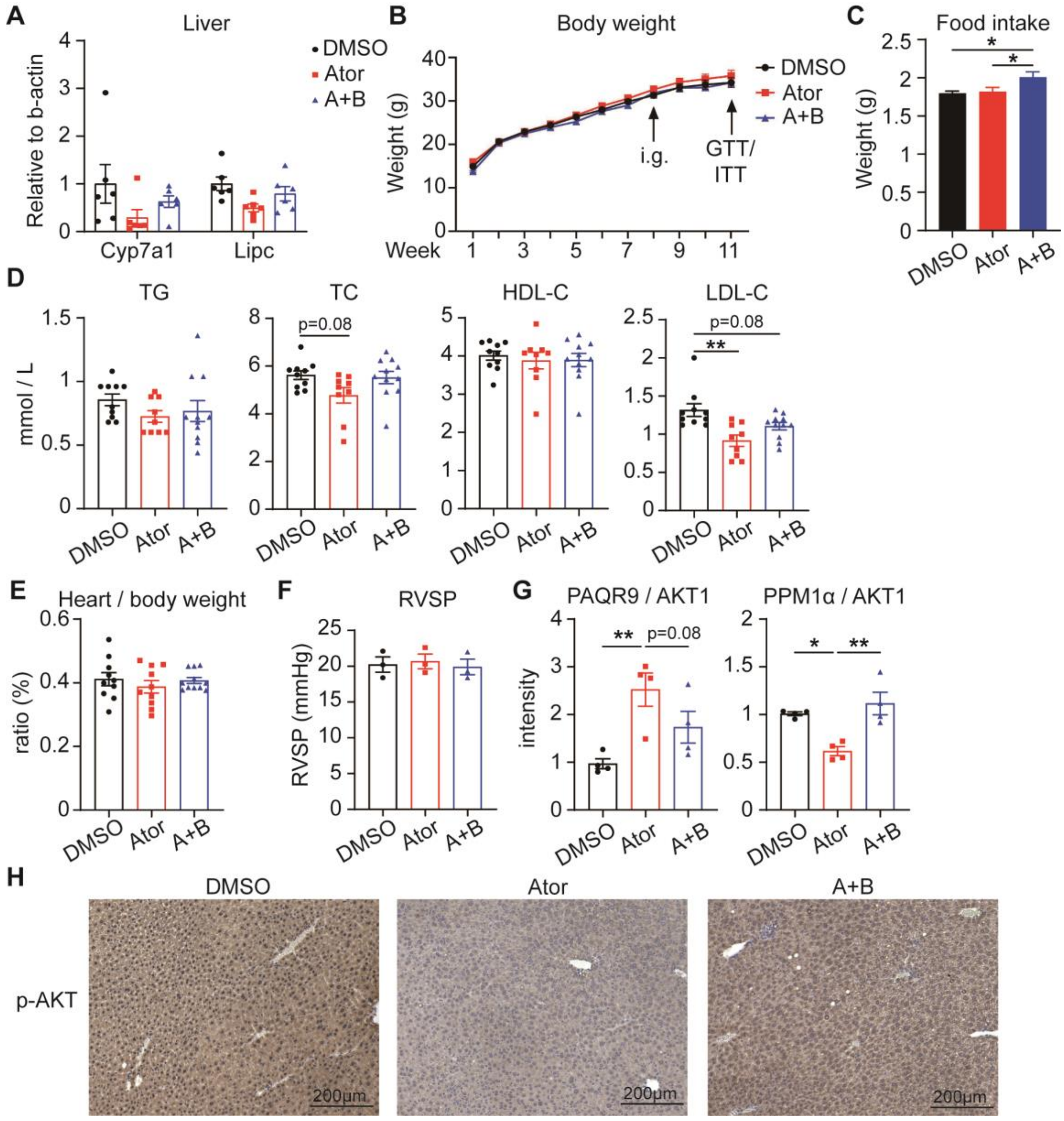


**Figure S7. Metabolic indicators of benflurex combined atorvastatin-treatment mouse model.**

**(A)** mRNA levels of *Cyp7a1* and *Lipc* in the liver of the mice as in **Figure 7A**. n=6 for each group.

**(B-C)** Body weight **(B)** and average food intake **(C)** of the mice.

**(D)** The levels of TG, TC, LDL-C and HDL-C in the serum of the mice.

**(E-F)** The heart weight ratio **(E)** and ventricular systolic blood pressure **(F)** of the mice.

For **B-E**, n=10 for group DMSO and group Ator, n=11 for group A+B. For **F**, n=3 for each group.

**(G)** Quantification of PAQR9 and PPM1α relative to total AKT1 of **Figure 7D**.

**(H)** Representative images of p-AKT1 staining of liver sections of the mice.

All the quantitative data were analyzed with one-way ANOVA and were shown as mean ±SEM. $^{*}P < 0.05$; $^{**}P < 0.01$.

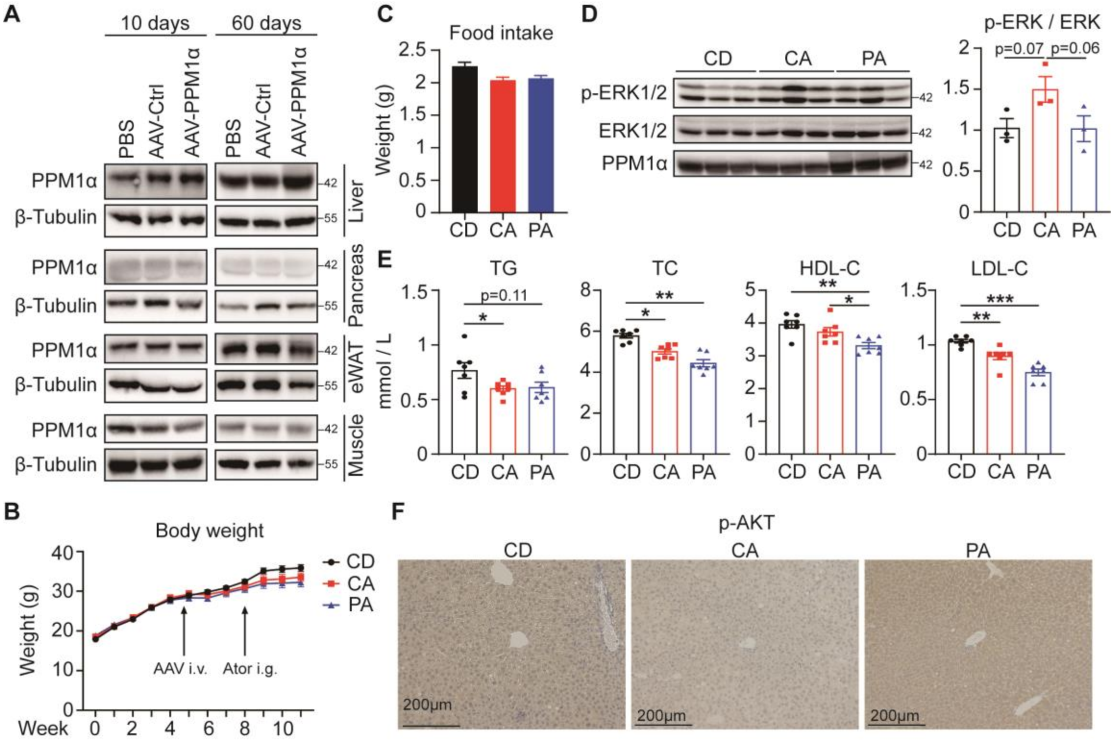


**Figure S8. Metabolic indicators of liver-specific PPM1α overexpression combined atorvastatin-treatment mouse model.**

**(A)** Western blotting to detect PPM1α in mouse liver, pancreas, eWAT and muscle. The mice were respectively sacrificed 10 days and 60 days after AAV i.v..

**(B-C)** Body weight **(B)** and average food intake **(C)** of the mice as in **Figure 7G**.

**(D)** Western blotting to detect ERK1/2 phosphorylation in the liver of the mice as in 7**G**. Quantification of p-ERK1/2 relative to ERK1/2 is shown in the right panel.

**(E)** The levels of TG, TC, LDL-C and HDL-C in the serum of the mice.

For **B,C, E**, n=7 for each group.

**(F)** Representative images of p-AKT1 staining of liver sections of the mice.

All the quantitative data were analyzed with one-way ANOVA and were shown as mean ±SEM. *$P < 0.05$; **$P < 0.01$; ***$P < 0.001$.

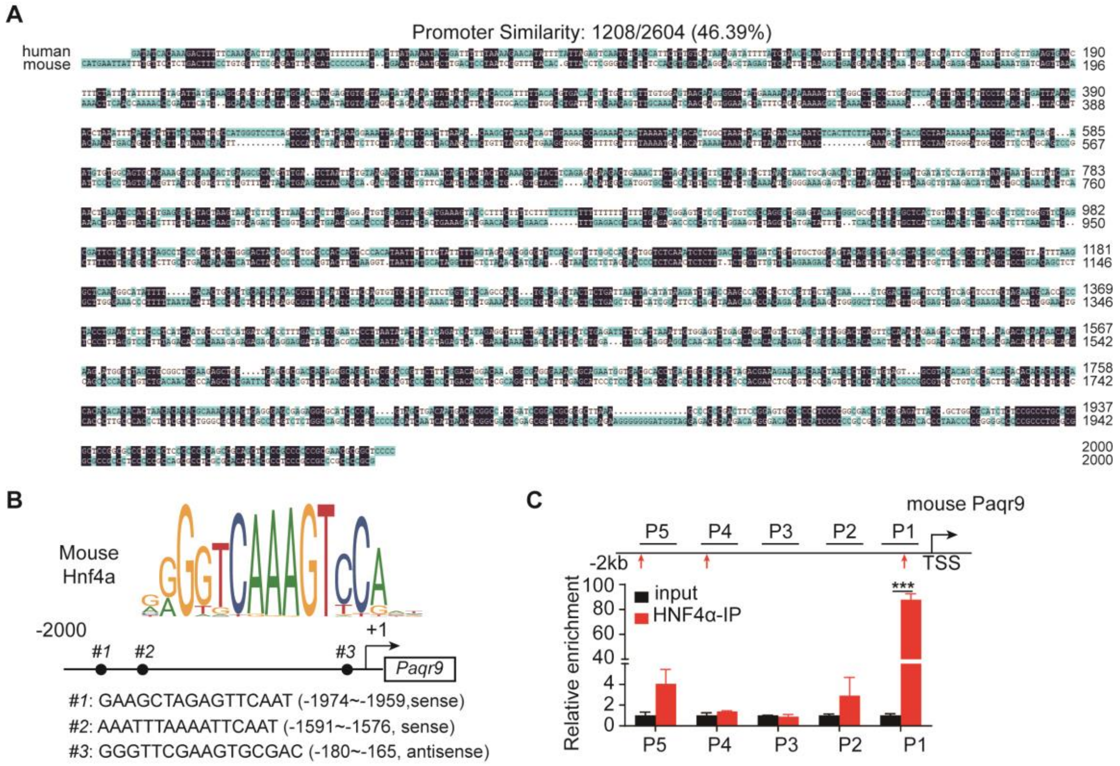


**Figure S9. The analysis of HNF4α targeting Paqr9 promoter in mouse.**

**(A)** The analysis of the homology of Paqr9 upstream sequences between human and mouse. Both sequences (-2000bp/+1bp) were input and aligned by DNAMAN.

**(B)** Mouse HNF4α motif analysis and potential binding sites at the *Paqr9* promoter.

**(C)** HNF4α ChIP-qPCR with different regions of *Paqr9* promoter in mouse primary hepatocytes. The red arrows pointed the predicted binding sites of HNF4α.

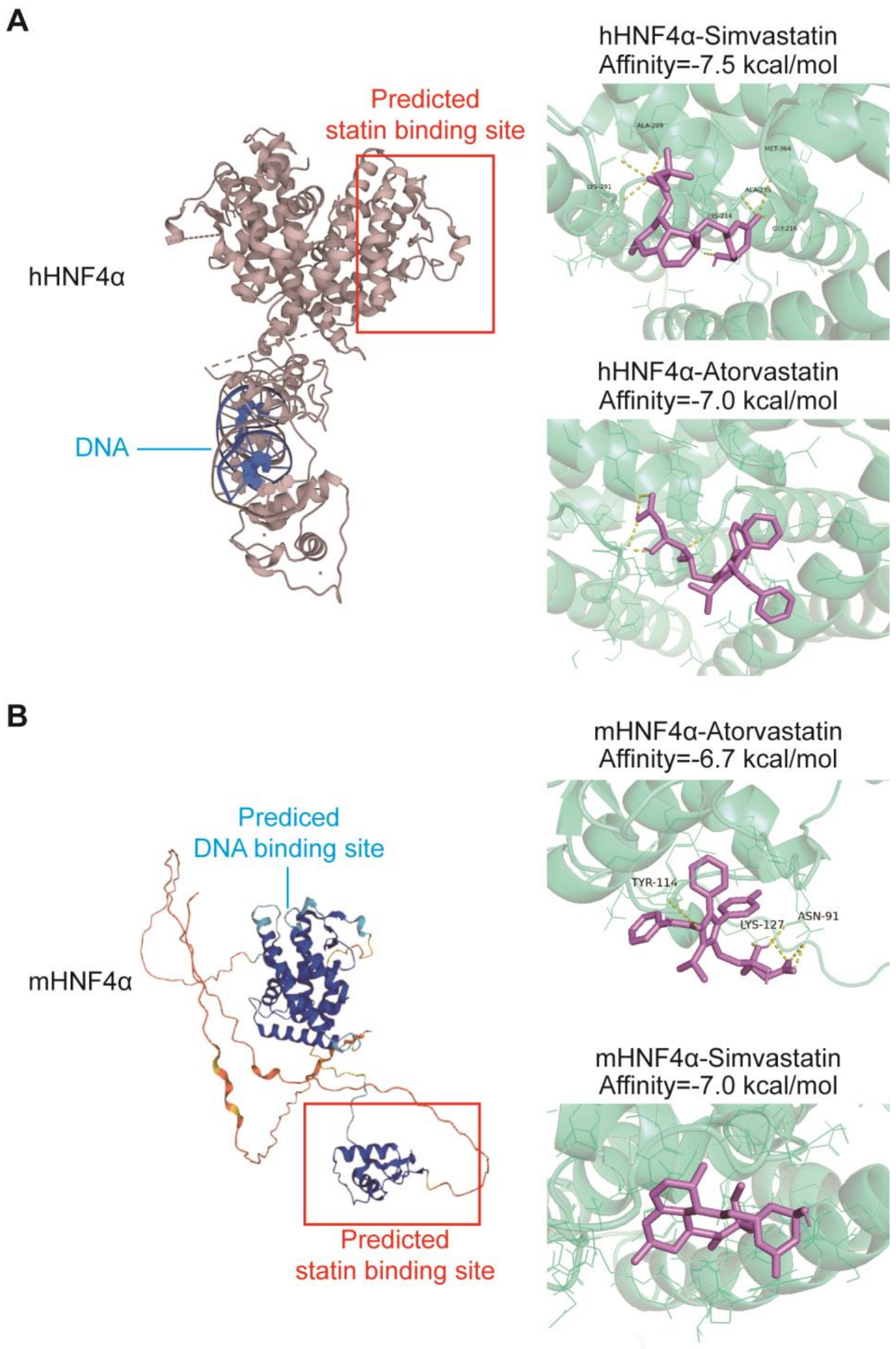


**Figure S10. Molecular docking of statins and HNF4α.**

**(A)** The structure of human HNF4α (left) and the binding mode of HNF4α with simvastatin and atorvastatin (right).

**(B)** The structure of mouse HNF4α (left) and the binding mode of HNF4α with simvastatin and atorvastatin (right).
